# Various Bone Disorders Affecting the Medically Underserved South Texas Region

**DOI:** 10.1155/joos/2858290

**Published:** 2025-05-21

**Authors:** Blake C. Martin, Manoj Peiris, Michael D. Sander

**Affiliations:** ^1^Department of Surgical Specialty and MSK, University of Texas Rio Grande Valley School of Medicine, Edinburg, Texas, USA; ^2^Department of Mathematics and Statistics, University of Texas Rio Grande Valley, Edinburg, Texas, USA

**Keywords:** bone condition, bone disease, bone disorder, underserved

## Abstract

Bone disorders can have a devastating impact on an individual's quality of life. The main objective of our study was to analyze various demographic disparities between various bone disorders in the demographically unique Rio Grande Valley region. We hypothesized that osteoporosis would be the most prevalent condition and that the demographics between the various conditions would vary depending on age, sex, BMI, and ethnicity but not on marital status. This was a retrospective chart review using the University of Texas Rio Grande Valley UTHealth electronic database from January 1, 2017, to January 1, 2024. Any relevant individuals seen at a UTRGV affiliated institution were included. We analyzed medical charts, via ICD-10 codes, of individuals who were diagnosed with osteoporosis, osteomalacia, rickets, Paget bone disease, and osteonecrosis. Bivariate and binary logistic regression analyses were performed to analyze the data. Hispanic ethnicity showed a decreased risk of bone disorder (estimate = −0.2814), bordering on significance (*p*=0.053). Female participants had significantly higher odds of bone disorder (estimate = 0.7861, *p* < 0.001). Obese individuals (estimate = −0.7837, *p* < 0.001) and overweight individuals had lower odds (estimate = −0.3328, *p*=0.047) of bone disorder. Underweight individuals showed higher odds of bone disorder (estimate = 0.9605, *p*=0.002). The odds of bone disorder increased with age (estimate = 0.0581, *p* < 0.001). Our results increase the knowledge of orthopedics in this region, and specifically, the bone disorders are discussed. The results may also allow physicians to better identify at-risk individuals in this community and others alike to improve the management of bone disorders. This study warrants further research on at-risk demographics to further improve the orthopedic knowledge and care of medically underserved individuals.

## 1. Introduction

### 1.1. Osteoporosis

Osteoporosis is a bone condition defined as low bone mineral density due to changes in bone microstructure, possibly leading to low-impact, fragility fractures [1–3]. These osteoporotic fractures can cause significant issues such as leading to a prominent decrease in quality of life, increasing morbidity, mortality, and disability [1, 3, 4]. Over two hundred million people have osteoporosis with the incidence rate increasing with age, with more than 70% of individuals over the age of 80 being affected [1, 3]. Millions of individuals are affected worldwide, with around nine million fractures occurring per year due to osteoporosis [1, 4]. Along with the significant effects on quality of life, the increasing age of the American population is estimated to triple the number of osteoporotic fractures [1]. In developed countries, approximately 2%–8% of males and 9%–38% of females are affected by this condition [1]. This condition is common in postmenopausal women, with more than 50% of postmenopausal White women sustaining an osteoporotic-related fracture, with around 33% of senior women being unable to return to independence [1, 3, 4]. Although the risk of an osteoporotic fracture is less in men, around 20%, the 1-year mortality in men who have these fractures is twice that of women [1, 3]. Some risk factors for osteoporosis include medications, preexisting diseases, increasing age, low bodyweight, smoking, family history of osteoporosis, White or Asian race, early menopause, low levels of physical activity, and a personal history of a fracture from a ground-level fall or minor trauma after the age of forty [1, 3, 4].

### 1.2. Osteomalacia

Osteomalacia, also known as “soft bone disease”, is a metabolic bone disorder that results from insufficient mineralization of bone tissue due to various mineral deficiencies such as vitamin D, calcium, or phosphate [5–7]. Weakened, softened bones, manifesting as bone pain, muscle weakness, and an increased fracture risk is what results from these deficiencies [5–7]. With vitamin D deficiency being the most common nutritional deficiency globally, osteomalacia presents significant concerns regarding bone health worldwide [5, 6]. Debilitating conditions are associated with this disorder, and thus, understanding osteomalacia is critical, as early recognition and appropriate treatment can prevent these issues [5, 7]. The true global incidence of osteomalacia is greatly underestimated as various risk factors exist, predisposing individuals to this condition [5, 7]. Some of these risk factors include dark skinned individuals, a lack of sun exposure, low socioeconomic status, poor diet, and individuals who frequently wear full-body clothing [5–7]. It is important to keep in mind that these risk factors vary depending on geographic location, cultural preferences, and ethnicity [5, 7].

### 1.3. Rickets

Rickets is a condition, acquired or inherited, that is characterized by a defect in mineralization of the epiphyseal plates [6, 8, 9]. Acquired rickets is the most common cause of rickets worldwide and is most commonly caused by deficiencies in vitamin D, calcium, and phosphorus [6, 8, 9]. The pathophysiology of mineral deficiencies makes it similar to osteomalacia; however, rickets occurs exclusively in children, whereas adults develop osteomalacia after the epiphyseal plate fusion [6, 8]. The condition can present in various ways from being asymptomatic to irritability, growth retardation, and sudden death [6, 8, 9]. The prevalence of rickets has been increasing in both developed and developing countries, although more common in the developing populations [6, 9]. In the United States, there has been a substantial increase in the incidence over the past 20 years, especially in higher latitude regions as restricted sunlight (UVB) exposure is a major factor [6, 8, 9].

### 1.4. Paget Bone Disease

Paget disease is a multifactorial, skeletal growth disorder with abnormalities such as unusual bone growth [10, 11]. The condition is due to excess osteoclastic activity followed by a compensatory increase in osteoblastic activity, giving rise to the formation of less compact, disorganized bone that is mechanically weaker, highly vascular, and more susceptible to fracture [10, 11]. It generally presents as diffuse pain and radiologically visible bone changes in the musculoskeletal system [10, 11]. It is a localized disease that does not spread to other bones; however, it can deteriorate the affected area and possibly progress to a potentially fatal sarcoma [10]. Paget disease is generally seen in individuals over the age of fifty [10, 11]. The worldwide prevalence has been reported to have decreased over the past 2 decades and is currently between 1.5% and 8.3% [10, 12]. It is common in Caucasians of northern European descent, is equally common in males and females, and affects approximately 1–3 million people in the United States [10, 11].

### 1.5. Osteonecrosis

Osteonecrosis, also known as avascular necrosis, is a degenerative bone condition characterized by the death of cellular components of the bone due to an interruption of the subchondral blood supply [13, 14]. This condition typically affects the epiphysis of long bones at weight-bearing joints and is most common at the femoral head (most common), knee, talus, and humeral head [13, 14]. If this disease advances, it could result in subchondral collapse, threatening the viability of the joint involved [13, 14]. Some risk factors include repetitive trauma, anatomy, glucocorticoids, alcohol misuse, blood dyscrasias, and autoimmune diseases [13, 14]. Of all hip arthroplasties in the United States, approximately 10 percent are due to osteonecrosis [13]. It generally affects ages 30 to 65, with males tending to be more affected [13, 14].

### 1.6. Study Significance and Aim

The underserved community that is the Rio Grande Valley (RGV), has a distinct demographic profile, with a high prevalence of chronic conditions such as diabetes and obesity, which warrants investigation of various health issues [15]. This region is also medically underserved, impoverished, and has a large population of undocumented immigrants [16, 17]. Furthermore, the machismo construct is another factor that may contribute to the health of this community as this may deter Hispanic males from seeking medical care due to it being perceived as feminine [18]. To our knowledge, there are no studies that have analyzed all these bone disorders in this medically underserved community. We conducted this study in order to further the knowledge of orthopedics in this unique population. The main objective of our study was to analyze the demographic disparities between osteoporosis, osteomalacia, rickets, Paget bone disease, and osteonecrosis in the RGV. We hypothesized that the frequency of osteoporosis would be the most common bone condition (of those analyzed) in the RGV and that the demographics between the various bone conditions would vary depending on age, sex, BMI, and ethnicity but not on marital status.

## 2. Methods

### 2.1. Study Design and Data Collection

This was a retrospective chart review, and IRB approval was obtained prior to starting this study. Data were gathered from the University of Texas RGV (UTRGV) UTHealth electronic database. Data collection included medical charts from January 1, 2017, to January 1, 2024. We collected and analyzed medical charts of individuals who were diagnosed with osteoporosis, osteomalacia, rickets, Paget bone disease, and osteonecrosis. The various disorders were obtained by using ICD-10 diagnosis codes. The codes were as follows (ICD-10 codes): osteoporosis (M80 and M81), osteomalacia (M83), rickets (E55.0), Paget bone disease (M88), and osteonecrosis (M87). For each patient, various demographics were collected, including BMI, sex, age at diagnosis, race/ethnicity, and marital status. Osteomalacia, rickets, and Paget bone disease were categorized together as “other disorders” due to the low frequency of diagnoses.

### 2.2. Inclusion and Exclusion Criteria

Individuals over the age of 85 were included as a group indicated as > 85 years of age. Individuals who were not seen by a UTRGV-associated institution were not included in the study. If there were duplications of an individual's medical chart, such as an individual having more than one appointment, the earliest date, the date the patient was diagnosed with any of the analyzed bone disorders, was included. If an individual was diagnosed with more than one of the bone disorders analyzed, all disorders were included and analyzed based on the date of diagnosis and demographics of the patient at the point in time of that specific diagnosis.

### 2.3. Data Analysis

Bivariate analyses were performed to assess the associations between each variable and the occurrence of any bone disorder (osteoporosis, osteomalacia, rickets, Paget bone disease, and osteonecrosis). For categorical variables (e.g., BMI, gender, and ethnicity), we used Chi-square tests to evaluate statistical independence with bone disorders. Fisher's exact test was used when expected cell counts were less than five to ensure accurate *p* value estimation.

To identify factors associated with any type of bone disorder (out of any three bone disorder types) and healthy controls, we performed a binary logistic regression analysis. The binary logistic regression model included having any type of bone disorder as the dependent variable and the demographics as predictors.

The logistic regression model was used to estimate the log odds of having any type of bone disorder, producing coefficients (log odds) for each predictor. Results were reported as odds ratios with 95% confidence intervals. Statistical significance was defined at the 0.05 level.

The binary logistic model fit was evaluated using residual deviance and Akaike information criterion (AIC) values. All analyses were performed with R statistical software (Version: 4.2.2 R Core Team, 2022).

## 3. Results

The data set highlights key demographic and clinical characteristics of individuals with bone disorders as well as those without such conditions. Males represent 32.2% of the population, while females make up 67.8%. The BMI distribution showed a high percentage of individuals missing BMI data (46.9%), followed by 25.3% classified as obese and 15.0% as overweight. Most participants are Hispanic or Latino (75.7%), with White individuals comprising 85.0% of the population, and 39.9% were married. These results are shown in Table 1.

**Figure 1:**
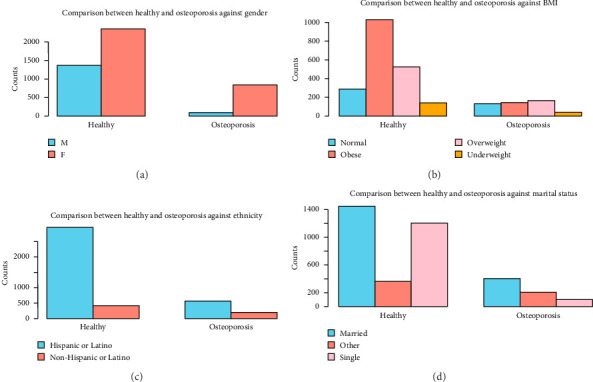
(a) Gender comparison between healthy and osteoporosis individuals. Healthy M: 1365, healthy F: 2352, osteoporosis M: 87, and osteoporosis F: 846. (b) BMI comparison between healthy and osteoporosis individuals. Healthy normal: 288, healthy obese: 1031, healthy overweight: 527, healthy underweight: 141, osteoporosis normal: 134, osteoporosis obese: 143, osteoporosis overweight: 168, and osteoporosis underweight: 41. (c) Ethnicity comparison between healthy and osteoporosis individuals. Healthy Hispanic: 2974, healthy non-Hispanic: 419, osteoporosis Hispanic: 570, and osteoporosis non-Hispanic: 202. (d) Marital status comparison between healthy and osteoporosis individuals. Healthy married: 1448, healthy single: 1207, healthy other: 363, osteoporosis married: 406, osteoporosis single: 102, and osteoporosis other: 206.

**Figure 2:**
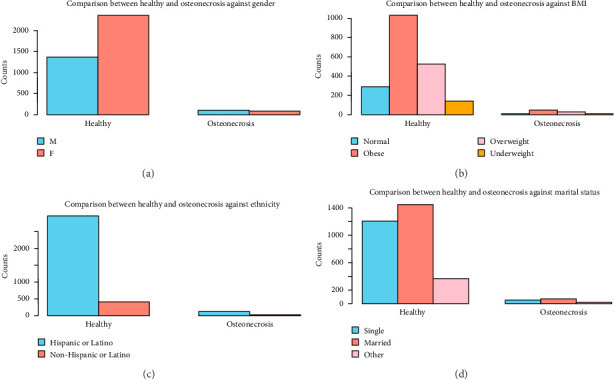
(a) Gender comparison between healthy and osteonecrosis individuals. Healthy M: 1365, healthy F: 2352, osteonecrosis M: 101, and osteonecrosis F: 84. (b) BMI comparison between healthy and osteonecrosis individuals. Healthy normal: 288, healthy obese: 1031, healthy overweight: 527, healthy underweight: 141, osteonecrosis normal: 9, osteonecrosis obese: 48, osteonecrosis overweight: 30, and osteonecrosis underweight: 7. (c) Ethnicity comparison between healthy and osteonecrosis individuals. Healthy Hispanic: 2974, healthy non-Hispanic: 419, osteonecrosis Hispanic: 121, and osteonecrosis non-Hispanic: 29. (d) Marital status comparison between healthy and osteonecrosis individuals. Healthy married: 1448, healthy single: 1207, healthy other: 363, osteonecrosis married: 71, osteonecrosis single: 52, and osteonecrosis other: 16.

**Figure 3:**
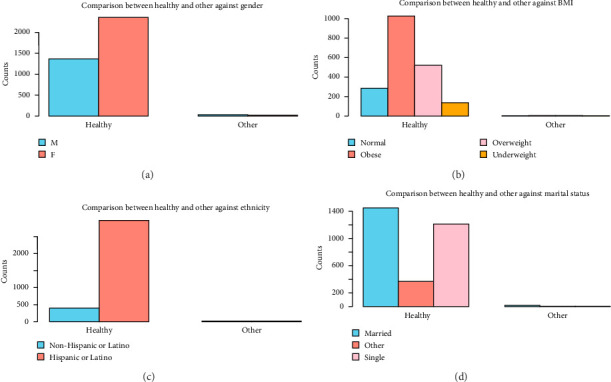
(a) Gender comparison between healthy and other bone disorder individuals. Healthy M: 1365, healthy F: 2352, other M: 13, and other F: 11. (b) BMI comparison between healthy and other bone disorder individuals. Healthy normal: 288, healthy obese: 1031, healthy overweight: 527, healthy underweight: 141, other normal: 1, other obese: 5, other overweight: 5, and other underweight: 0. (c) Ethnicity comparison between healthy and other bone disorder individuals. Healthy Hispanic: 2974, healthy non-Hispanic: 419, other Hispanic: 13, and other non-Hispanic: 7. (d) Marital status comparison between healthy and other bone disorder individuals. Healthy married: 1448, healthy single: 1207, healthy other: 363, other married: 13, other single: 2, and other: 2.

**Figure 4:**
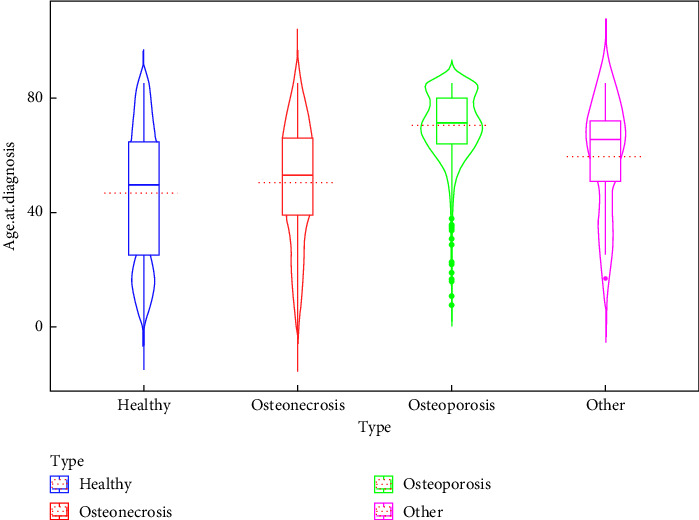
Violin box plot between age at diagnosis and bone disorder types. The red dash lines represent the mean age for the corresponding group.

Clinically, the most common condition observed was osteoporosis (19.2%), followed by osteonecrosis (4.7%) and other types (1.5%). A significant majority (76.4%) are classified as healthy (without bone disorders), indicating that a smaller subset of the population has active bone disorders. These results are shown in Table 1. Patient characteristics associated with each bone disorder are shown in Figures 1, 2, 3, 4.

### 3.1. Osteoporosis

Gender, BMI, ethnicity, and marital status comparison between healthy and osteoporosis individuals (see Figure 1).

### 3.2. Osteonecrosis

Gender, BMI, ethnicity, and marital status comparison between healthy and osteonecrosis individuals (see Figure 2).

### 3.3. Other Disorders

Gender, BMI, ethnicity, and marital status comparison between healthy and other bone disorders (see Figure 3).

### 3.4. Bivariate Analysis

The following bivariate analysis results can be seen in Table 2.

  Gender
• Osteoporosis females had significantly higher odds (OR = 5.633) compared to males (*p* < 0.001).
• Osteonecrosis females had significantly lower odds (OR = 0.482) compared to males (*p* < 0.001).
  BMI
• Compared to individuals with a normal BMI, those who were obese (OR: 0.298, *p* < 0.001), overweight (OR: 0.685, *p* < 0.001), or underweight (OR: 0.627, *p* < 0.001) had lower odds. The BMI was not significantly associated with the odds of osteonecrosis or other disorders.
  Race
• No significant differences were observed across racial groups for any type of disorder (*p*=0.103, *p*=0.409, and *p*=0.243).
  Ethnicity
• Ethnicity showed significant associations across all conditions. Hispanic or Latino individuals had lower odds of osteoporosis (OR: 0.397, *p* < 0.001), osteonecrosis (OR: 0.586, *p*=0.017), and other disorders (OR: 0.259, *p*=0.006) compared to non-Hispanic or Latino individuals.
  Marital status
• Osteoporosis: Married (OR: 3.313) and other marital statuses (OR: 6.702) were associated with significantly higher odds compared to single individuals (*p* < 0.001) under.
• Married (OR: 5.088) and other marital statuses (OR: 3.322) had higher odds of other disorders (*p*=0.031).

### 3.5. Age at Diagnosis

Healthy vs. osteoporosis showed a highly significant difference with osteoporosis occurring at later ages (*Z* = −29.92, adjusted *p* value (P.adj) < 0.0001). Osteonecrosis vs. osteoporosis showed a strong difference with osteoporosis occurring at a later age (*Z* = −12.02, P.adj < 0.0001). Healthy vs. other disorders showed a modest significant difference with other disorders occurring at later ages (*Z* = −2.82, P.adj = 0.028).

The violin plot (Figure 4) shows the distribution of age at diagnosis across four groups based on the “Type” variable: healthy, osteonecrosis, osteoporosis, and other. The shape of each violin indicates the density of data points, with wider sections reflecting higher densities. For the healthy group, the distribution is relatively narrow, with a symmetric shape, as suggested by the alignment of the median and the central region of the violin. In the osteonecrosis group, the violin is narrower, indicating less variation in age, and the median is centrally located, suggesting a balanced distribution. The osteoporosis group displays a wider spread in ages, with the violin shape suggesting potential skewness and a higher median age compared to other groups. For the other group, the violin shows a narrower distribution, with the median centrally positioned, reflecting symmetry in the distribution. Overall, the graph highlights the differences in the central tendencies (median) and variability in the age of diagnosis across the groups.

### 3.6. Binary Logistic Regression Analysis

When we considered all variables together, we had 2085 subjects (excluding any missing values), and they were used for the binary logistic regression model building purposes. Hispanic or Latino ethnicity showed a decreased risk of bone disorder (estimate = −0.2814), bordering on significance (*p*=0.053). Female participants had significantly higher odds of bone disorder compared to males (estimate = 0.7861, *p* < 0.001). Obese individuals had lower odds (estimate = −0.7837, *p* < 0.001), and overweight individuals had lower odds (estimate = −0.3328, *p*=0.047) of bone disorder, compared to those with a normal BMI. Underweight individuals showed significantly higher odds of bone disorder (estimate = 0.9605, *p*=0.002). Age at diagnosis was a strong positive predictor (estimate = 0.0581, *p* < 0.001), indicating that the odds of bone disorder increased with age.

## 4. Discussion

After controlling for the numerous variables in our study, our results show that Hispanic or Latino ethnicity has a decreased risk of bone disorder (estimate = −0.2814), bordering on significance (*p*=0.053), indicating a potential protective effect. The current literature states that this is true when comparing Hispanics to non-Hispanic Whites, and Asians as Hispanics have a lower prevalence of osteoporosis compared to these groups [19]. However, non-Hispanic Blacks have a lower prevalence of osteoporosis compared to Hispanics [19]. The limited ethnical information in our data could help explain this difference as we were not able to separate the individuals in the non-Hispanic groups into their own ethnical/racial groups. With all non-Hispanic individuals being combined into a single group, we were unable to analyze the specifics of non-Hispanic groups. However, Hispanic individuals seem to have a marginally significant decreased risk of obtaining osteoporosis. Regarding osteonecrosis, we were unable to find any studies analyzing the relationship between Hispanic and non-Hispanic individuals to compare our results to. Further studies should be conducted to analyze osteonecrosis protective factors that could be at play in this Hispanic population to explain this finding. Regarding the other bone disorders analyzed in this study, Paget disease is more common in Caucasians, osteomalacia in darker-skinned ethnicities, and rickets not showing a relationship with ethnicity. Our results do not agree with the literature on Paget disease or rickets but somewhat agree with the literature on osteomalacia as Hispanics tend to have relatively dark skin [5–8, 10, 11]. Further studies may focus on the relationship between ethnicity and Paget disease as well as ethnicity and rickets in this study population to determine the factors that could be involved.

Regarding gender, female participants have significantly higher odds of bone disorder compared to males (estimate = 0.7861, *p* < 0.001), suggesting that sex is a strong predictor. In terms of osteoporosis, the current literature agrees with females having an increased risk of osteoporosis [1, 3, 4]. This may be due to females having smaller bones, thinner bones, and less bone mass compared to males [1, 3, 4]. The decreased level of sex hormones such as estrogen when females hit menopause is also a strong risk factor for females to develop osteoporosis, while males do not have this major risk factor [1, 4]. Regarding osteonecrosis and the other bone disorders analyzed in this study, the current literature does not show a difference in risk based on sex [5–11, 13, 14]. An explanation for this is that most of our patients in this study had osteoporosis which could increase the effect of sex on bone disorders overall, showing an increased risk in females. However, when analyzed individually, although gender did not significantly affect “other” bone disorders, being female showed a decreased risk of osteonecrosis. It is possible that various factors that increase the risk of osteonecrosis are present in males of the RGV population. Further studies investigating osteonecrosis should be conducted to further investigate this finding.

Regarding the BMI, obese individuals (estimate = −0.7837, *p* < 0.001) and overweight individuals (estimate = −0.3328, *p*=0.047) have lower odds of bone disorder, compared to those with a normal BMI. Underweight individuals show significantly higher odds of bone disorder (estimate = 0.9605, *p*=0.002). The current literature states that lower BMI individuals have an increased risk for osteoporosis, but that BMI does not have a significant effect on the risk of obtaining any of the other bone disorders analyzed in this study [1, 5–11, 13, 14]. As mentioned in the previous paragraph, the large number of individuals in our study who had osteoporosis could skew the effect of the BMI overall on the bone disorders analyzed. However, when analyzed separately, the BMI did not show a significant effect on osteonecrosis and the other disorders studied which agrees with the current literature [5–11, 13, 14].

Age at diagnosis is a strong positive predictor (estimate = 0.0581, *p* < 0.001), indicating that the odds of bone disorder increase with age. This agrees with the current literature on osteoporosis and Paget disease [1, 3, 4, 10, 11]. However, osteonecrosis typically affects middle to older adults (30–65), rickets affects children, and osteomalacia affects adults of various ages [5, 6, 8, 13, 14]. Again, the large number of individuals with osteoporosis in our study could skew the effect of age on bone disorders as a whole. The group of osteonecrosis individuals having the second most patients could also factor into this as it tends to affect middle to older individuals. However, the group of “other” bone disorders showed to have a significant increase in age compared to healthy individuals when analyzed alone. Paget disease and osteomalacia made up most individuals in this group (19/24). This helps explain why increased age was shown to have an increased risk of these disorders.

Marital status had no effect on the overall risk of having a bone disorder when adjusting for the variables in this study. However, when analyzed individually, married and other individuals had an increased risk of osteoporosis and “other” bone disorders compared to single individuals. An explanation for this could be that individuals who have a significant other or some other form of social support are more likely to be encouraged by their social support to seek medical help when symptoms or concerns arise. However, this relationship with marital status was not significant for osteonecrosis. We were unable to find any articles in the current literature analyzing marital status and these disorders. Further studies may focus on the effect of marital status and/or social support on bone disorders.

There are limitations to this study. Data were only collected from the UTRGV UTHealth electronic databases; therefore, individuals who sought care at an institution not affiliated with UTRGV were not considered in this study. This study may not be generalizable to the entire South Texas population because of the data source. Most of the patients analyzed in this study were Hispanic, which indicates that our results may not be generalizable to the United States or another nation's general population whose ethnicity landscape differs from the one in our study. This South Texas population is a poverty stricken, medically underserved area with a large population of undocumented immigrants and individuals without health insurance [16, 17]. The study results may not be universally applied due to this unique patient community.

Future studies should focus on obtaining data from a more generalizable source, obtaining a larger study size, and including more demographic factors that could play a role in the development of these various bone disorders to increase study validity and generalizability. Furthermore, separation and individual analysis of the various bone disorders should be performed.

## 5. Conclusion

Our results increase the knowledge of orthopedics in this region, specifically regarding the bone disorders discussed. The results may also allow physicians to better identify at-risk individuals in this community and others alike to improve the management of bone disorders. This study warrants further research on at-risk demographics to further improve the orthopedic knowledge and care of medically underserved individuals.

## Figures and Tables

**Table 1:** Patient demographics.

Variable	*N* = 4859 (100%)
Gender	
§ Male	1566 (32.2%)
§ Female	3293 (67.8%)
BMI	
§ Underweight	189 (3.9%)
§ Normal	432 (8.9%)
§ Overweight	730 (15.0%)
§ Obese	1227 (25.3%)
§ NA	2281 (46.9%)
Ethnicity	
§ Hispanic or Latino	3678 (75.7%)
§ Non-Hispanic or Latino	657 (13.5%)
§ Patient declined	524 (10.8%)
Race	
§ White	4129 (85.0%)
§ Other (including Black African Americans)	220 (4.5%)
§ Patient declined	510 (10.5%)
Marital status	
§ Single	1363 (28.0%)
§ Married	1938 (39.9%)
§ Other	587 (12.1%)
§ Patient declined	971 (20.0%)
Type	
§ Osteoporosis	933 (19.2%)
§ Osteonecrosis	185 (1.9%)
§ Other	24 (0.4%)
§ Healthy	3717 (76.4%)

**Table 2:** Summary of Chi square/Fisher's exact test.

		Osteoporosis		Osteonecrosis		Other disorders	
		OR (95% CI)	*p* value	OR (95% CI)	*p* value	OR (95% CI)	*p* value
Gender	Male	Ref	< 0.001	Ref	< 0.001	Ref	0.120
	Female	5.633 (4.499, 7.138)		0.482 (0.358, 0.650)		0.492 (0.214, 1.113)	

BMI	Normal	Ref	< 0.001	Ref	0.471	Ref	0.663^∗^
	Obese	0.298 (0.228, 0.391)		1.468 (0.745, 3.253)		0.801 (0.177, 6.076)	
	Overweight	0.685 (0.524, 0.897)		1.785 (0.872, 4.100)		1.564 (0.345, 11.883)	
	Underweight	0.627 (0.415, 0.933)		1.594 (0.549, 4.433)		1.082 (0.034, 13.472)	

Race	Other	Ref	0.103	Ref	0.409	Ref	0.243^∗^
	White	0.747 (0.541, 1.045)		0.700 (0.380, 1.451)		0.416 (0.118, 2.855)	

Ethnicity	Non-Hispanic or Latino	Ref	< 0.001^∗^	Ref	0.017	Ref	0.006
	Hispanic or Latino	0.397 (0.329, 0.481)		0.586 (0.391, 0.906)		0.259 (0.104, 0.703)	

Marital status	Single	Ref	< 0.001	Ref	0.772	Ref	0.031^∗^
	Married	3.313 (2.642, 4.189)		1.137 (0.790, 1.647)		5.088 (1.384. 35.467)	
	Other	6.702 (5.155, 8.762)		1.030 (0.562, 1.788)		3.322 (0.345, 32.010)	

*Note:* This shows the results for assessing the relationship between the three types of bone disorders vs. healthy control variations and the demographic variables. 95% CI represents the 95% confidence interval for the crude odds ratio. The *p* values based on Fisher's exact tests are denoted by ^∗^.

## Data Availability

The data that support the findings of this study are available from the University of Texas Rio Grande Valley electronic medical records. Restrictions apply to the availability of these data, which were used under license for this study. Data are available from the authors with the permission of the University of Rio Grande Valley School of Medicine Institutional Review Board.
